# Shoulder Pain Associated With Multiple Arteriovenous Malformations Successfully Treated With Embolization

**DOI:** 10.7759/cureus.33669

**Published:** 2023-01-11

**Authors:** Frank A Cusimano, Martyna Czarnik, Anthony Watkins, Adam Tonis, David A Greuner

**Affiliations:** 1 Department of Research, NYC Surgical Associates, New York, USA; 2 Internal Medicine, University of Miami Miller School of Medicine, Jackson Memorial Hospital, Miami, USA; 3 Kidney Transplant Program, Tampa General Hospital, Tampa, USA; 4 Department of Surgery, University of South Florida Morsani College of Medicine, Tampa, USA; 5 Department of Surgery, NYC Surgical Associates, New York, USA

**Keywords:** gelfoam, traumatic avm, vascular embolization, arteriovenous malformations, transarterial embolization, transvenous embolization, capillary venous malformation

## Abstract

A 44-year-old male presented with left upper extremity and shoulder pain with worsening functional impairment after years of repetitive use, overtraining, and multiple injuries from weightlifting and mixed martial arts. Imaging showed no obvious injury or ligamentous deformity other than mild osteoarthritis (OA) of the left glenohumeral joint. Duplex ultrasonography (US) revealed four arteriovenous malformations (AVMs) surrounding the shoulder joint and left upper extremity. The vasculature was mapped via angiography through a transradial approach. Initial treatment included transarterial embolization of two AVMs off the axillary artery and branching anterior circumflex humeral artery. Secondary treatment included embolization of two lesions months later via direct puncture, one through a transvenous approach and the second through direct transmalformation cannulation, via the nidus, near the clavicle and posterior scapular lateral border. Treatment resulted in significant improvement in pain and range of motion. Follow-up assessments revealed improvement in overall symptoms, recovered function, and return to exercise and competitive mixed martial arts. This case highlights the value of duplex ultrasonography, embolization, and transarterial and transvenous approaches for the treatment of AVM-associated extremity or joint pain.

## Introduction

Peripheral arteriovenous malformations (AVM) are congenital or acquired vascular anomalies characterized by aberrant flow, abnormal connections between vasculature, and abnormal flow resistance [1,4]. While the pathophysiology of AVMs remains unknown, most are genetic or acquired. It is not uncommon for AVMs to be dormant at birth but manifest symptoms later in life when the lesions become large enough to cause hemodynamic irregularities [5]. Acquired AVMs may manifest idiopathically or be triggered by trauma, hormones, tumor growth, or infection [6]. Elevated expression of endothelial progenitor cells and vasculogenesis are thought to contribute to AVM manifestation and progression [7]. Like most vascular formations, AVMs grow proportionally with the flow demand and growth rate of the specific tissue of the affected patient, often worsened by pregnancy, puberty, and trauma [8,9].

AVMs are difficult to diagnose, manage, and treat. Hemodynamic changes are associated with pain, pressure, and decreased function and can be associated with hemorrhage, aneurysm, muscle failure, and tissue destruction, depending on the location and nature of the lesion [10]. Arteriovenous malformations have a prevalence of 1%, with the extremities being the most common site of these vascular lesions outside the head and neck [11]. Symptoms include pain, cutaneous discoloration, pulsatile masses, local warmth, bone overgrowth, weeping, bleeding or changes in symptoms with exertion [8,12]. Peripheral tissues may be affected by hypertrophy, atrophy, necrosis, and ulceration [13]. Overall, patients with symptomatic AVMs report a lower health-related quality of life [14,15].

Treatment depends on the angiographic pattern and location of the lesion, with surgery or endovascular intervention being the standard. Endovascular embolization is a less invasive, catheter-based intervention that is a safe alternative to traditional resection with a small risk of nontarget embolization, arterial damage, contrast-induced nephropathy, and puncture site bleeding [16]. Surgical resection has been associated with a high complication rate, high rate of recurrence, extensive intraoperative bleeding, and long-term tissue damage [17,18].

Direct cannulation and embolization is a treatment for patients with symptomatic arteriovenous malformations. Here, we report the successful treatment of a 44-year-old male with four symptomatic arteriovenous malformations of the left upper extremity associated with left shoulder pain and decreased function after years of repetitive use injury and blunt trauma. Diagnosis was made via duplex ultrasonography, lesions were mapped via angiography, and the patient was treated with transarterial embolization with tris-acryl polymer embosphere microspheres and direct transvenous and transmalformation cannulation embolization with gelfoam, reporting near-complete improvement in pain, symptoms, and functioning.

## Case presentation

A 44-year-old male with a past medical history of osteoarthritis, back pain, and hypercholesterolemia presented with pain, occasional swelling, and decreased range of motion to the left shoulder. Pain was described as a dull ache, occasional burning pain after exercise with limited use of the left shoulder. Pain was localized to the shoulder joint, the lateral deltoid, trapezius, and near the clavicle. Pain was exacerbated by excessive movement, lifting weights over 5 pounds, exercise increasing circulatory demand, and mixed martial art movements requiring the upper extremities. Pain was partially relieved with ice and rest after not using the shoulder for a few days. Physical examination revealed local swelling and pain to the deltoid and trapezius with palpation. On initial examination, patient had a positive drop arm test, belly test, and Jobe test, and forced abduction test, but a negative Neers test, Hawkins test, apprehension test, drawer test, sulcus test, speed test, Yergason test, empty can test, speed test, Roos test, and compression test. The patient had been trialing conservative treatment for three years, including over-the-counter acetaminophen, ibuprofen, ice, heat, physical therapy, rest, acupuncture, and muscle energy with mild temporary relief. Pain always returned or worsened after circulatory demand increased.

Diagnostic radiographic imaging revealed osteoarthritis of the glenohumeral joint. Magnetic resonance imaging revealed intact supraspinatus, infraspinatus, subscapularis, and teres minor. Normal subacromial and subdeltoid bursa. No muscle atrophy. Biceps tendon intact was without evidence of tendinopathy or tenosynovitis. Mild degenerative changes of the acromioclavicular joint without evidence of separation, effusion, or ligamentous injury. Normal subacromial and subcoracoid arch and normal glenoid labrum without evidence of a superior labral anterior posterior (SLAP) tear of the glenoid labrum. No evidence of fractures or skeletal or soft tissue infections. Normal axilla without adenopathy. Doppler ultrasonography of the upper left extremity revealed one symptomatic high-flow arteriovenous malformation near the surgical neck of the humerus and three possible AVMs surrounding the left shoulder, near the posterior deltoid, acromioclavicular joint, and the clavicle. After informed consent and a complete discussion of risks, benefits, alternatives, and the expected postoperative course, including the possible need for additional procedures, the patient was taken for angiography of the left upper extremity with intraoperative doppler ultrasonography. A bubble study was obtained preoperatively, ruling out the possibility of paradoxical embolus and patent foramen ovale (PFO).

**Figure 1:**
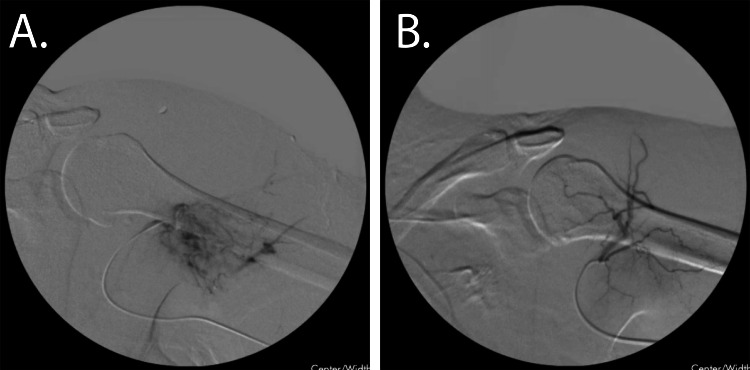
Transarterial Arteriovenous Malformation (AVM) Embolization A. Fluoroscopic imaging of the left upper extremity with visualization of an AVM off the anterior circumflex humeral artery cannulated transarterially from the radial artery. B. Post embolization, improved flow can be seen to the anterior circumflex humeral artery and complete cessation of flow to the AVM.

**Figure 2:**
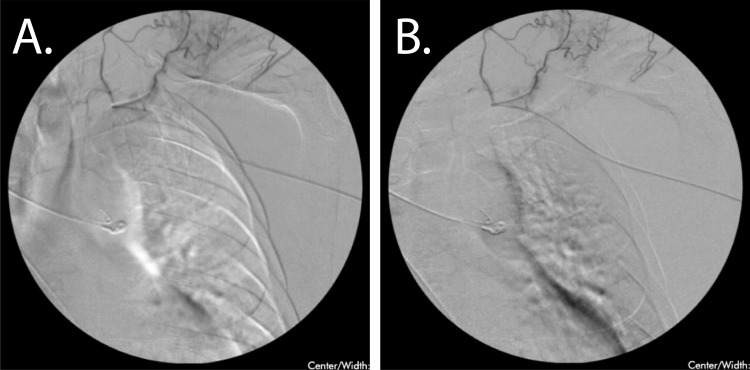
Transarterial Arteriovenous Malformation (AVM) Embolization A. Fluoroscopic imaging of the left upper extremity with visualization of an AVM off an aberrant malformation off the axillary artery cannulated transarterially from the radial artery. B. Post embolization, trace improvement was seen with improved blushing but no cessation of flow to the identifiable vessels.

The patient was brought into the operating room and prepped and draped in the customary sterile fashion. The limb was mapped out, distal runoff vessels were checked and a suitable cannulation location was chosen. A transarterial approach was used with ultrasound-guided left radial artery cannulation using a 4 French micropuncture sheath. The guidewire was advanced from the radial artery to the brachial, then to the axillary artery. Subselective cannulation of secondary branches was performed to verify location and positioning. Two regions, one off the anterior circumflex humeral artery (Figure 1) and one aberrant malformation off the axillary artery (Figure 2), were subselectively cannulated to decrease flow rates and then embolized with Terumo HydroPearl embospheres (Terumo, Somerset, NJ, USA). On-table, real-time color duplex scanning post-procedure was performed to ensure cessation of flow in the target lesion off the anterior circumflex humeral artery and patent native distal runoff. There was no visualization of non-target embolization or cessation of flow to surrounding vasculature. Although four abnormal vessels were originally identified on ultrasound, the decision was made to treat the first two malformations and re-evaluate during follow-up.

On post-op follow-up, 24 hours after embolization, patient reported mild pain at the cannulation site, but no interarticular discomfort or pain in the arm or at the glenohumeral joint. At two-week follow-up, patient reported improvement in overall symptoms but still complained of discomfort near the supraspinatus and trapezius radiating to the glenohumeral joint. Since initial embolization, he resumed mixed martial arts reporting improvement up to 90% of his reported baseline. The decision was made to watch and monitor before repeat intervention.

**Figure 3:**
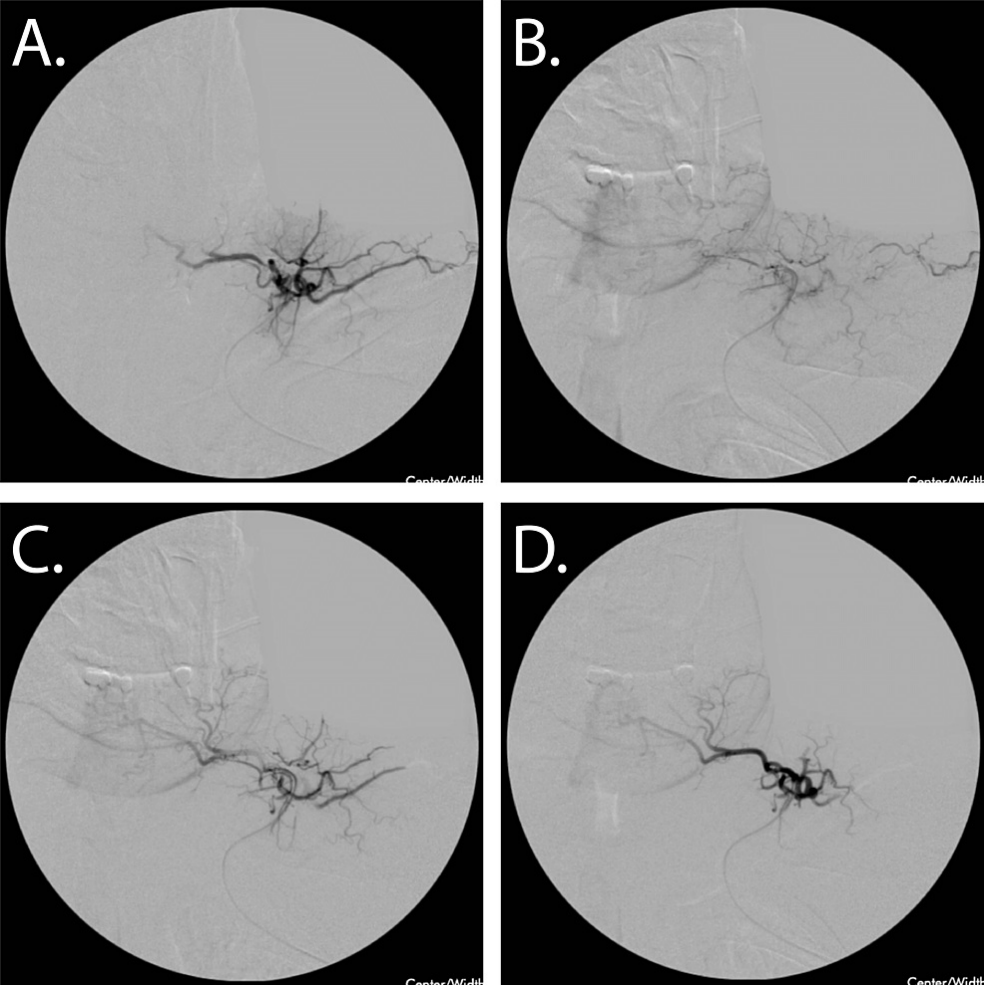
Supraclavicular Transvenous Arteriovenous Malformation (AVM) Embolization A. Fluoroscopic imaging of the AVM off the subclavian vein proximal to the bifurcation of the cephalic vein transvenously cannulated. B. Fluoroscopic imaging of the feeding tributaries feeding the venous supply. C-D. Post embolization, patent flow of the feeding vasculature with improved flow from the cephalic tributaries. Overall improvement was seen with improved blushing but no cessation of flow to the identifiable vessels.

**Figure 4:**
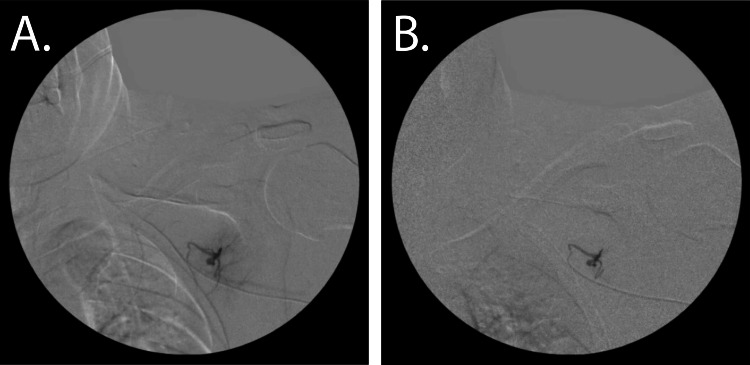
Posterior Transmalformation Arteriovenous Malformation (AVM) Embolization A. Fluoroscopic imaging of the AVM. B. Post embolization, patent flow of the feeding vessel with improved blushing and cessation of flow to the AVM.

Three months after initial treatment, patient was brought back in for additional treatment as he reported continued symptoms though a change in location from initial presentation. On preoperative ultrasonography, he was noted to have two monophasic, moderate flow lesions, one supraclavicular nidus with a velocity of 44 cm/s superior to the clavicle not consistent with the transscapular artery, and a second posterior nidus with a velocity measuring 21 cm/s along the lateral border of the scapula near the intersection of the deltoid and teres minor. After a complete discussion of risks, benefits, alternatives, and expected postoperative course, patient was brought to the operating room and prepped and draped in the customary sterile fashion. A transvenous direct cannulation approach was performed using a 4 French micropuncture catheter under real-time ultrasound guidance near the clavicle (Figure 3). Contrast was used to verify cannulation and identification of the malformation and location. Embolization was performed using small aliquots of gelfoam with continuous reassessment of the anatomy between doses using color flow duplex and angiography until flow cessation of the neovascularized area. Post procedure, color duplex scanning was performed to ensure cessation of flow to the target lesion. Next, the posterior nidus was identified and a transmalformation direct cannulation approach was performed near the lateral border of the scapula (Figure 4). The AVM was embolized using small aliquots of gelfoam with mild improvement in blushing and flow. Ultrasound mapping of the upper left extremity was performed to ensure patent flow. No evidence of localized compartment syndrome was identified. The patient noted significant improvement in pain immediately following the procedure.

At 24-hour follow-up and at two-week follow-up, patient reported near complete resolution of initial symptoms. He reported some mild discomfort at the start of exercise but was now back to weightlifting and mixed martial arts. Six-month follow-up reviewed continued results without reoccurrence or new symptoms. Long-term follow-up is planned.

## Discussion

Above, we report the successful treatment of four symptomatic arteriovenous malformations of the left upper extremity with transarterial, transvenous, and transmalformation direct cannulation embolization. These treatments offer a less invasive option with a low risk of complication. Given AVM unpredictability, successful treatment often requires a multimodal approach with more than one treatment session. Non-target embolization is a well-documented complication of AVM-related endovascular procedures. Care was taken to prevent complications by modifying the approach for each lesion [11].

For patients with acquired AVMs, the distribution of blood flow can cause areas of high-flow, turbulence, or enlarged draining veins through intervening low-pressure niduses. With such an incalculable behavior pattern, AVMs are difficult to diagnose, manage, and treat. The abnormality between the venous and arterial circulation results in altered blood pressure [3,19].

Future research should focus on using validated treatment outcomes for vascular malformations, such as the Lymphatic Malformation Function (LMF) instrument questionnaire for patients with vascular malformations [20]. Additionally, studies are needed to provide more data on the treatment and management of patients with high-flow AVMs and the various approaches to treatment.

## Conclusions

In the present study, we successfully treated a patient’s chronic shoulder pain with both transarterial, transvenous, and direct transmalformation cannulation embolization. The patient reported a high patient satisfaction score, including immediate pain relief following the procedure and satisfaction with the procedural experience and results. The patient self-reported an improvement of pre-interventional symptoms following the procedure. Direct puncture cannulation and embolization reduces the risk of perioperative complications for patients, improves recovery time, and minimizes operating time and operating costs. Embolization is a safe and effective treatment for symptomatic peripheral AVMs after initial conservative therapies fail.
